# 

**Published:** 1931

**Authors:** 


### The Medical Library of the University of Bristol,

WITH WHICH IS INCORPORATED

**The Library of the Bristol Medico-Chirurgical Society.**

---

*The following donations have been received since the publication of the list in September,* 1931.

December, 1931.

| | |
|---|---|
| Dr. D. R. Acheson (1) .. .. .. | 4 volumes. |
| Blacker Library of Zoology (2) .. .. | 1 volume. |
| Dental Board of the United Kingdom (3) | 1 ,, |
| Glasgow Royal Maternity and Women's Hospital (4) .. .. .. .. | 1 ,, |
| Prof. E. W. Hey Groves (5).. .. .. | 1 ,, |
| London School of Hygiene and Tropical Medicine (6) .. .. .. .. | 1 ,, |
| Michell Clarke Memorial Fund (7) .. .. | 24 volumes. |
| Munro Smith Memorial Fund (8) .. .. | 10 ,, |
| Royal College of Surgeons of England (9) .. | 1 volume. |

Unbound periodicals have also been received from Professor E. W. Hey Groves and Dr. Carey F. Coombs.

### THE ONE HUNDRED AND THIRTY-EIGHTH LIST OF BOOKS.

The figures in round brackets refer to the figures after the names of the donors. The books to which no such figures are attached have either been bought from the Library Fund or received through the *Journal.*

**Bailey, H.** .. .. .. *Demonstrations of Physical Signs in Clinical Surgery* .. .. .. .. .. .. .. 3rd Ed. 1931

**Bainbridge, F. A.** .. *The Physiology of Muscular Exercise.* 3rd Ed. (8) 1931

**Barritt, W. B., and A. T.** *Simple Handbook of Dental Materia Medica and Therapeutics* .. .. .. 2nd Ed. 1923

**Bayliss, W. M.** .. .. *Principles of General Physiology* 4th Ed. (8) 1927

**Blair, V. P., and Ivy, R. H.** *Essentials of Oral Surgery* .. .. (7) 1926

**Bowlby, A. A., and Andrewes, F. W.** *Surgical Pathology and Morbid Anatomy* .. .. .. .. .. .. 8th Ed. (7) 1930

**Boyd, M. F.** .. .. *An Introduction to Malariology* .. .. (7) 1930

**Boyd, W.** .. .. .. *Pathology of Internal Disease* .. .. (7) 1931

**Bulleid, A.** .. .. *A Text-book of Bacteriology for Dental Students* 1926

**Burchard, H. H., and Inglis, O. E.** *Text-book of Dental Pathology and Therapeutics* .. .. .. .. .. .. (7) 1927

**Castellani, A.** .. .. *Climate and Acclimatization* .. .. .. (8) 1931

**Cavallo, T.** .. .. *Complete Treatise of Electricity* .. .. (1) 1777

**Colyer, F.** .. .. .. *Abnormal Conditions of the Teeth of Animals in Their Relationship to Similar Conditions in Man* .. .. .. .. .. .. .. .. (3) —

**Cox, J. M.** .. .. *Practical Observations on Insanity* 2nd Ed. (1) 1806

**De Lee, J. B.** .. .. *Principles and Practice of Obstetrics* 5th Ed. (7) 1930

**Economo, C. von** .. *Encephalitis Lethargica: its Sequel and Treatment* .. .. .. .. .. .. .. .. 1931

**Fenton, W. J., and Burrell, L. S. T.** *Diseases of the Chest* .. .. (7) —

**Findlay, L.** .. .. .. *Rheumatic Infection in Childhood* .. .. (7) 1931

**Garrod, A. E.** .. .. *Inborn Factors in Disease* .. .. .. (7) 1931

**Hamilton, R.** .. .. *Observations on Scrophulous Affections* .. (1) 1791

**Harrison, L. W.** .. *Diagnosis and Treatment of Venereal Diseases in General Practice* .. .. .. .. .. (7) 1931

**Hart, B.** .. .. .. *Psychology of Insanity* .. .. .. .. (7) 1930

**Hippisley, Sir J., Bart.** *Speech on the petition of the Roman Catholics in Ireland* .. .. .. .. .. 2nd Ed. (1) 1810

**Hopewell-Ash, E.** .. *An A.B.C. of Medical Hypnosis* .. .. .. —

**Hornibrook E. A.** .. *Restoration Exercises for Women* .. .. .. 1931

**Hughlings-Jackson, J.** *Selected Writings.* Vol. I. .. .. .. (7) 1931

**Kligler, I. J.** .. .. *Epidemiology and Control of Malaria in Palestine* .. .. .. .. .. .. .. .. 1930

**Lewis, T.** .. .. .. *Clinical Disorders of the Heart Beat* 6th Ed. (7) 1925

**Lloyd, W.** .. .. .. *Hay-fever, Hay-Asthma* .. .. .. .. .. 1931

**Lyle, H. W., and De Souza, D.** *Manual of Physiology* 3rd Ed. (8) 1930

**McCarthy, L.** .. .. *Histopathology of Skin Diseases* .. .. (7) 1931

**Marshall, A. M., and Hurst, C. H.** *A Junior Course of Practical Zoology* .. .. .. .. .. 11th Ed. (8) 1930

**Masters, W. E.** .. *The Alcohol Habit and its Treatment* .. 1931

**Mathews, A. P.** .. *Physiological Chemistry* .. .. 5th Ed. (8) 1930

*Medical Evidence before the Royal Commission on Licensing*, 1930 .. 1931

**Mennell, J.**.. .. .. *Backache* .. .. .. .. .. .. .. .. .. (7) 1931

**Nattrass, F. J.** .. .. *The Commoner Nervous Diseases* .. .. .. 1931

**Newth, G. S.** .. .. *A Text-book of Inorganic Chemistry.* New and Enlarged Ed. 1926

**Plimmer, R. H. A.** .. *Practical Organic and Bio-Chemistry.* New Ed. (8) 1926

**Poynton, F. J., and Schlesinger, B.** *Recent Advances in the Study of Rheumatism* .. .. .. .. .. .. .. (7) 1931

**Pryde, J.** .. .. .. *Recent Advances in Bio-Chemistry* 3rd Ed. (8) 1931

**Singer, C.** .. .. .. *A Short History of Biology* .. .. .. (8) 1931

**Smith, S., and Glaister, J.** *Recent Advances in Forensic Medicine* (7) 1931

**Stewart, A. W.** .. *Recent Advances in Physical and Inorganic Chemistry* .. .. .. .. 6th Ed. 1930

*System of Bacteriology in Relation to Medicine.* Vol. IX. .. .. 1931

**Thomson, A., Miles, A., and Wilkie, D. P. H.** *Manual of Surgery,* 3 vols. .. .. .. .. .. .. 8th Ed. (5) 1931

*Tuberculosis : its Treatment and Cure with the Help of Umckaloabo* (Stevens). By an English Physician .. —

**Walker, J.** .. .. .. *Organic Chemistry for Students of Medicine* 2nd Ed. 1919

**Walton, A. J.** .. .. *Text-book of Surgical Dyspepsias* 2nd Ed. (7) 1930

**Ward, M. L.** .. .. *American Text-book of Operative Dentistry* 6th Ed. (7) —

**Williams, S.** .. .. *Experimental History of Road Water in Wiltshire* .. .. .. .. .. .. .. .. 1731

**Wilson, S. A. K.** .. *Modern Problems in Neurology* .. .. (7) 1928

**Winnicott, D. W.** .. *Clinical Notes on Disorders of Childhood* .. 1931

**Winton, F. R., and Bayliss, L. E.** *Human Physiology* .. .. (8) 1930

**Wood, C. A.** .. .. *An Introduction to the Literature of Vertebrate Zoology* (2) .. .. .. .. .. .. .. .. 1931

**Wright, S.** .. .. .. *Applied Physiology* .. .. .. 4th Ed. 1931

### TRANSACTIONS, REPORTS, JOURNALS, ETC.

Annals of the Pickett-Thomson Research Laboratory. Vol. 6. December, 1930 .. .. .. .. .. .. .. .. .. .. .. .. .. (7) 1930

Glasgow Royal Maternity and Women's Hospital—Medical Report for 1930 .. .. .. .. .. .. .. .. .. .. .. .. .. (4) 1931

International Health Year Book, 1929 .. .. .. .. .. .. .. (7) 1929

London School of Hygiene and Tropical Medicine—Collected Addresses and Laboratory Studies. Vol. 6 .. .. .. .. .. .. (6) 1929–30

Progressive Medicine. Vol. 3. September, 1931 .. .. .. .. .. 1931

Royal College of Surgeons of England Calendar, 1931 .. .. (9) 1931

Saint Thomas's Hospital Reports. Vol. 53 .. .. .. .. .. .. .. 1929

Surgical Clinics of North America, August, Vol. 2, Nos. 4 and 5. August and October, 1931 .. .. .. .. .. .. .. .. .. .. 1931

### Local Medical Notes

EXAMINATION RESULTS.

**University of Bristol.**—Students of the University have recently passed the following examinations :—

M.B., Ch.B.—*Supplementary First Examination. Pass :* J. C. D. Harris, Ursula G. Hewitt. *In Chemistry* (*Completing Examination*) *:* G. W. Vowles.

M.B., Ch.B. (*Part I.*).—*Final Examination. Pass :* S. B. Adams, A. J. Board, Violet Fry, Winifred M. Hill (with distinction in Materia Medica, Pharmacy, Pharmacology and Therapeutics), Aldyth D. Jones, Gwladys R. Llewelyn (with distinction in Pathology), H. R. Pearse (with distinction in Pathology, Forensic Medicine and Toxicology), Frances E. Powell, Mary G. Thomas.

M.B., Ch.B. (*Part II.*).—*Final Examination. Pass :* F. W. A. Fosbery, J. A. Kersley, A. J. B. Miall, S. C. Wake. *In Group I.* (*Completing Examination*) *:* O. J. P. Bollon. *In Group I. only :* A. C. Price.

L.D.S.—*Supplementary Preliminary Science Examination. Pass : In Physics and Biology only :* D. E. Royal.

L.D.S.—*Final Professional Examination. Pass :* N. Miller, H. L. Saunders.

L.D.S.—*Second Professional Examination. Pass : In Dental Mechanics and Dental Metallurgy* (*Completing Examination*) *:* I. R. R. Eskell. *In Dental Materia Medica* (*Completing Examination*) *:* R. L. Royal. *In Dental Mechanics and Dental Metallurgy only :* J. H. G. Smith.

**Conjoint Board.**—M.R.C.S., L.R.C.P. *Pass : In Elementary Biology :* Myrtle M. Baxter, Susie C. Sampson. *In Chemistry and Physics :* M. Thompson. *In Anatomy and Physiology :*

S. Roberts. *In Physiology :* L. E. Sealy. *In Medicine :* C. J. N. Davis.* *In Midwifery :* R. G. C. G. Carlson, C. N. Royal, G. M. Evans. *In Pathology :* F. E. Fletcher, N. C. Coombs, Enid A. Taylour, J. F. H. Eagles, A. M. D. Davies. *In Pathology and Midwifery :* A. L. Eyre-Brook, H. F. M. Finzel, O. E. L. Sampson, G. T. Bevir, Frances N. Salisbury. *In Surgery :* J. Hughes, T. J. Ashley.*

L.D.S., R.C.S.—*Final Examination. Pass : Part I. :* A. E. Hawkins. *Part II. :* E. G. Dowland, W. A. Duly, A. M. Watson (Royal Dental Hospital).

**Royal College of Surgeons.**—F.R.C.S. *Final :* H. E. Harris (M.B. Camb.), T. H. Berrill (M.B., Ch.B. Bristol), N. C. Tanner (M.B., Ch.B. Bristol), G. F. Langley (M.B., Ch.B., Bristol).

**University of Liverpool.**—DIPLOMA OF TROPICAL MEDICINE. H. J. H. Spreadbury, M.B., Ch.B. (Bristol), M.R.C.S., L.R.C.P.

* Qualified.

### Index

Abdomen—
See under Appendicitis, acute.
Adams, A. W. and Birrell, J. A.—A case of congenital obstruction of the bile-duct, 215.
Anæsthesia—
The partnership between anæsthesia and surgery.—A. L. Flemming, 233.
Appendicitis, acute—
A review of seven hundred cases.—H. Chitty, 167.

Bile-duct—
A case of congenital obstruction.—J. A. Birrell and A. W. Adams, 215.
Birrell, J. A., and Adams, A. W.—A case of congenital obstruction of the bile-duct, 215.
Book Reviews, 57, 155, 219, 283.
Bristowe, H. C.—Sterilization of the unfit, 189.
Brock, L. G.—The modern outlook on mental health, 97.
Broncho-pneumonia—
Cyanosis in the fatal broncho-pneumonia of influenza.—F. E. Fletcher, 277.
Bush, G. B.—Silicosis: its radiology and pathology, 259.

Carleton, H. H.—Some disorders of movement and their mechanism, 135.
Chambers, E. R.—Differential diagnosis of primary iritis in the early stages, 51.
Children—
See under Heart.
Chitty, H.—A review of seven hundred cases of acute appendicitis, 167.
Coombs, C. F.—The work of the University Centre of Cardiac Research, 179.
Cross, F. R.—Obituary, 226.
Cyanosis—
Cyanosis in the fatal broncho-pneumonia of influenza.—F. E. Fletcher, 277.

Ear, external—
Congenital abnormalities. — E. Watson-Williams, 273.
Editorial Notes—
Annual Medical Dinner, 290.
Bristol Medico-Chirurgical Society's President's Badge, 160.
Colston Research Society.—Annual Dinner, 161.
Colston Medical Research Fellowships, 289.
Edward Jenner's books for the Medical Library, 291.
General Hospital—New Out-Patient Department, 65.
*Lancet* Commission on Nursing, 224.
National Council for Women—Second Biennial Conference, 66.
Obituaries—
F. R. Cross, 226.
Sir Vernon Wills, 65.
Paul Bush Gold Medal, 160.
Queen Victoria Jubilee Convalescent Home, 223.
Royal Society of Medicine, Section of Anæsthetics—Meeting in Bristol, 159.
Endocrine Glands—
Relation of gynæcology to.—D. C. Rayner, 1.
Flemming, A. L.—The partnership between anæsthesia and surgery—Long Fox Memorial Lecture, 233.

Fletcher, F. E.—Cyanosis in the fatal broncho-pneumonia of influenza, 277.

General Hospital—
New Out-Patient Department, 65.
Glands—
See under Endocrine Glands.
Goitre—
See under Tetany.
Gynæcology—
Relation to glands of internal secretion.—D. C. Rayner, 1.

Hæmaturia—
Hæmaturia in the pre-eruptive stage of measles.—F. G. Jenkins, 275.
Heart—
Necrosis of the myocardium.—H. Rogers, 35.

Heart (*continued*)—
Congenital heart disease as seen in elementary school children.—C. B. Perry, 41.
The work of the University Centre of Cardiac Research.—C. F. Coombs, 179.
Hernia—
Strangulated.—N. E. F. Kemm, 151.

Influenza—
Cyanosis in the fatal broncho-pneumonia of influenza.—F. E. Fletcher, 277.
Intestines—
Some intestinal malformations and their clinical significance.—A. L. Taylor, 113.
Iris, inflammation—
Differential diagnosis of primary iritis in the early stages.—E. R. Chambers, 51.
Iritis—
See under Iris, inflammation.

Jenkins, F. G.—Hæmaturia in the pre-eruptive stage of measles, 275.

Kemm, N. E. F.—Strangulated hernia, 151.

Library Notes—
Accessions, 71, 165, 230, 296.
Local Medical Notes—
Committee's Medals: J. J. J. Giraldi (gold), G. F. Langley (silver), 74.
Examination Results, 73, 165, 231, 299.
Long Fox Memorial Lecture: A. L. Flemming, The partnership between anæsthetics and surgery, 74.
Radcliffe Prize—W. H. Bradley, 74.
Royal College of Physicians, London—Goulstonian Lectures delivered by M. Critchley on "Neurology of old age," 74.
Long Fox Memorial Lecture: The partnership between anæsthesia and surgery.—A. L. Flemming, 233.

Maclean, Sir E. J.—Some reminiscences and a forecast, 83.
Measles—
Hæmaturia in the pre-eruptive stage of measles.—F. G. Jenkins, 275.
Mental Defectives—
Sterilization.—H. C. Bristowe, 189.
Mental Hygiene—
The modern outlook on mental health.—L. G. Brock, 97.
Movements—
Some disorders of movement and their mechanism.—H. H. Carleton, 135.
Muscles—
See under Movements.
Myocardium—
See under Heart.

National Council for Mental Hygiene—Second Biennial Conference at Westminster, 66.
Nixon, J. A.—Silicosis, 253.

Obituaries—
F. R. Cross, 226.
Sir Vernon Wills, 65.

Parathyroidectomy—
See under Tetany.
Perry, C. B.—Congenital heart disease as seen in elementary school children, 41.
Pneumoconiosis—
Silicosis.—J. A. Nixon, 253.
Silicosis: its radiology and pathology. G. B. Bush, 259.
Pneumonia—
See under Broncho-Pneumonia.
Pregnancy—
Ectopic.—R. S. S. Statham and H. L. Shepherd, 15.

Rayner, D. C.—Relation of gynæcology to the glands of internal secretion, 1.
Rogers, H.—Necrosis of the myocardium, 35.

Shepherd, H. L., and Statham, R. S. S.—Ectopic pregnancy, 15.
Silicosis—
See under Pneumoconiosis.
Societies—
Bristol Medico-Chirurgical Society:
Ectopic gestation.—R. S. S. Statham, 67.
Remarks by
A. T. F. Rowley.
A. Rendle Short.
W. S. V. Stock.
A. L. Flemming.
H. Rogers.
D. A. Alexander.
Lily A. Baker.
S. J. H. Griffiths.
Striæ atrophicæ.—D. A. Alexander—case, 68.
Silicosis of lung.—G. B. Bush—X-rays, 68.
Necrosis of myocardium.—H. Rogers, 68.

Societies (*continued*)—
Congenital heart disease as seen in elementary school children.—C. B. Perry, 68.
Remarks by
C. E. K. Herapath.
R. Clarke.
A. L. Taylor.
A. F. Alford.
Spinal anæsthesia.—A. W. Adams, 69.
Remarks by
W. A. Gornall.
N. E. F. Kemm.
D. Wood.
S. J. H. Griffiths.
W. S. V. Stock.
C. Corfield.
Some reminiscences and a forecast.—Sir E. J. Maclean, 69.
Remarks by
A. J. M. Wright.
C. F. Walters.
D. A. Alexander.
A. E. J. Lister.
R. H. Parry.
Some disorders of movement and their mechanism. — H. H. Carleton, 70.
Remarks by
J. V. Blachford.
R. G. Gordon.
F. Bodman.
W. P. Kennedy.
Officers, 75.
Past Presidents, 76.
Subscribers, 77.
Intestinal abnormalities and their clinical significance.—A. L. Taylor, 162.
Remarks by
H. Rogers.
A. D. Fraser.
G. R. Scarff.
Sterilization of the unfit.—H. C. Bristowe, 163.
Annual General Meeting, 292.

Societies (*continued*)—
Silicosis.—Two papers by Drs. G. B. Bush and J. A. Nixon, 295.
Remarks by
F. J. A. Mayes.
H. H. Carleton.
W. P. Kennedy.
D. A. Alexander.
F. G. Bergin.
J. R. Charles.
Treatment of anterior poliomyelitis.—R. G. Gordon and M. F. Forrester - Brown — Cinematograph film, 296.
British Association of Dermatology and Syphilology — Annual Meeting, 232.
Statham, R. S. S., and Shepherd, H. L.—Ectopic pregnancy, 15.
Sterilization of the unfit. — H. C. Bristowe, 189.
Remarks by
R. J. A. Berry.
C. F. Walters.
J. R. Charles.
J. O. Symes.
R. G. Gordon.
H. H. Carleton.
A. G. Morison.
W. R. Dawson.
A. N. P. Milner.
F. Bodman.
P. King.

Taylor, A. L.—Some intestinal malformations and their clinical significance, 113.
Tetany following thyroid operations.—D. Wood, 205.
Thyroid—
See under Tetany.

University Centre of Cardiac Research, The work of.—C. F. Coombs, 179.

Watson - Williams, E. — Congenital abnormalities of the external ear, 273.
Wills, Sir Vernon.—Obituary, 65.
Wood, D.—Tetany following thyroid operations, 205.

J. W. Arrowsmith Ltd
Publishers Bristol

### Publications Received

From GEORGE ALLEN & UNWIN LTD.:

*The Neural Energy Constant.* By JOHN BOSTOCK, M.B., B.S., D.P.M., M.R.C.S., L.R.C.P. Price 6s.

From CASSELL & CO. LTD.:

*The Cause of Cancer.* By W. E. GYE, M.D., and W. J. PURDY, M.B. Price 30s.

*Modern Medical Treatment.* By E. BELLINGHAM-SMITH, M.D., F.R.C.P., and A. FEILING, F.R.C.P. Price 30s.

*Radiology in Relation to Modern Jurisprudence.* By S. G. SCOTT, M.R.C.S., L.R.C.P., D.M.R. & E. Price 7s. 6d.

From WILLIAM HEINEMANN (MEDICAL BOOKS) LTD.:

*Fertility and Sterility in Marriage.* By TH. H. VAN DE VELDE, M.D. Price 25s.

*Clinical Notes on Disorders of Childhood.* By D. W. WINNICOTT, M.A., M.R.C.P. Price 10s. 6d.

*Towards National Health.* By J. ANTHONY DELMEGE, O.B.E., M.R.C.S., L.R.C.P., D.P.H. Price 21s.

*Sex Hostility in Marriate.* By TH. H. VAN DE VELDE, M.D. Price 17s. 6d.

From THE LANCET LTD.:

*Guy's Hospital Reports.* Vol. lxxxi, No. 3. July, 1931. Price 12s. 6d.

From H. K. LEWIS & CO. LTD.:

*Rheumatoid Arthritis and its Treatment.* By VINCENT COATES, M.C., M.A., M.D., M.R.C.P., and LEO DELICATI, L.M.S.S.A. Price 6s.

*A Manual of General Medical Practice.* Second Edition. By W. S. SYKES, M.A., M.B., B.Ch. Price 7s. 6d.

*Minor Surgery.* Second Edition. By L. R. FIFIELD, F.R.C.S. Price 12s. 6d.

*Management of Abdominal Operations.* By RODNEY H. MAINGOT, F.R.C.S. Price 7s. 6d.

*Bendien's Diagnostic Methods for Cancer and Principles of Treatment.* By A. A. MILLER, M.D. Price 3s. 6d.

*Handbook of Sanitary Law for the Use of Candidates for Public Health Qualifications.* Eleventh Edition. By B. BURNETT HAM, M.D., D.P.H. Price 7s. 6d.

From OXFORD UNIVERSITY PRESS:

*Thomson and Miles' Manual of Surgery.* Eighth Edition. Vols. ii. and iii. By A. MILES, M.D., LL.D., F.R.C.S., and D. P. D. WILKIE, M.D., F.R.C.S. Price 12s. 6d. per vol.

From KEGAN, PAUL, TRENCH, TRUBNER & CO. LTD.:

*Phylaxis.* By G. BILLARD, M.D. Price 9s.

*The Genesis of Cancer.* By W. S. HANDLEY, M.S., F.R.C.S. Price 21s.

From JOHN WRIGHT & SONS LTD.:

*An Index of Treatment by Various Authors.* Edited by R. HUTCHISON, M.D., F.R.C.P. Price 42s.

*The British Journal of Surgery.* Vol. XIX., No. 74. October, 1931. Price 12. 6d.

*Emergency Surgery,* Vol. ii. By HAMILTON BAILEY, F.R.C.S. Price 25s.

